# Oculomotor capture by search-irrelevant features in visual working memory: on the crucial role of target–distractor similarity

**DOI:** 10.3758/s13414-020-02007-0

**Published:** 2020-03-12

**Authors:** Rebecca M. Foerster, Werner X. Schneider

**Affiliations:** 1 Center for Interdisciplinary Research (ZIF), Bielefeld University, Bielefeld, Germany; 2 Neuro-cognitive Psychology, Department of Psychology, Bielefeld University, P.O. Box 100131, D-33501 Bielefeld, Germany; 3 ‘Cognitive Interaction Technology’ Cluster of Excellence (CITEC), Bielefeld University, Bielefeld, Germany

**Keywords:** Oculomotor capture, Visual working memory, Object-based attention, Visual search, Target–distractor similarity

## Abstract

When searching for varying targets in the environment, a target template has to be maintained in visual working memory (VWM). Recently, we showed that search-irrelevant features of a VWM template bias attention in an object-based manner, so that objects sharing such features with a VWM template capture the eyes involuntarily. Here, we investigated whether target–distractor similarity modulates capture strength. Participants saccaded to a target accompanied by a distractor. A single feature (e.g., shape) defined the target in each trial indicated by a cue, and the cue also varied in one irrelevant feature (e.g., color). The distractor matched the cue’s irrelevant feature in half of the trials. Nine experiments showed that target–distractor similarity consistently influenced the degree of oculomotor capture. High target–distractor dissimilarity in the search-relevant feature reduced capture by the irrelevant feature (Experiments 1, 3, 6, 7). However, capture was reduced by high target–distractor similarity in the search-irrelevant feature (Experiments 1, 4, 5, 8). Strong oculomotor capture was observed if target–distractor similarity was reasonably low in the relevant and high in the irrelevant feature, irrespective of whether color or shape were relevant (Experiments 2 and 5). These findings argue for involuntary and object-based, top-down control by VWM templates, whereas its manifestation in oculomotor capture depends crucially on target–distractor similarity in relevant and irrelevant feature dimensions of the search object.

**Electronic supplementary material:**

The online version of this article (10.3758/s13414-020-02007-0) contains supplementary material, which is available to authorized users.

Imagine that a friend asks you to help him search for a cup from a set of cups in a cupboard filled with many differently shaped and colored cups. While pointing to a cup on the table, he says, “I am searching for a cup from the same set as this one. They all have the same shape, but are different in color.” You would need to tune your top-down control in order to preferentially let objects capture your attention that resemble the exemplar’s shape, while ignoring all objects matching the exemplar’s color. But is that possible? Can we ignore a specific feature of a relevant search object in order not to be captured by this feature? If so, which processing characteristics might influence such an attentional biasing process?

Substantial evidence supports the claim that we can tune our attention in a top-down fashion in order to search for a specific target on the basis of a search template stored in visual working memory (VWM) so that environmental objects matching the template will preferably be selected (Conci, Müller, & von Mühlenen, 2013; Folk, Remington, & Johnston, 1992; Hollingworth, 2012; Wolfe, 1994; Woodman & Chun, 2006). Within the biased competition framework, this top-down guided selection should be achieved by giving higher attentional weights to objects matching target features (Bundesen, 1990; Desimone & Duncan, 1995; Duncan & Humphreys, 1989). How effective might a VWM template be in allowing top-down selection?

Does any VWM object (e.g., also an item that is a distractor for an ongoing task) bias competition in a search process? Some studies investigated this issue by means of a dual-task paradigm that consisted of a search task performed during the retention interval of a VMW task. Some studies show that if the distractor of the search task matched a VWM representation, visual attention was captured obligatorily, even if participants knew about the distracting nature of the memory content (Downing, 2000; Olivers, 2009; Olivers, Meijer, & Theeuwes, 2006; Pashler & Shiu, 1999; Soto, Heinke, Humphreys, & Blanco, 2005; Soto, Hodsoll, Rotshtein, & Humphreys, 2008; Soto & Humphreys, 2009; Soto, Humphreys, & Heinke, 2006). However, other studies found no obligatory capture (Downing & Dodds, 2004; Olivers et al., 2006; Sala & Courtney, 2009; Woodman & Luck, 2007), and further studies found that a distractor that predictably matched VWM content improved search performance, arguing that such a matching distractor could more efficiently be ignored than a distractor that had no features in common with the VWM content—a mechanism called template rejection (Arita, Carlisle, & Woodman, 2012; Han & Kim, 2009; Woodman & Luck, 2007).

To reconcile the opposing results, Olivers and colleagues (Olivers, Peters, Houtkamp, & Roelfsema, 2011) suggested that VWM representations can be kept in either an active or an accessory state. Only active items should bias attention in an ongoing task. Because the memory items are not needed for the interim search task in the aforementioned dual-task studies, they can be kept in an accessory or passive format during search and will not bias attention. However, for strategical reasons, participants might have kept VWM items in an active format in some studies—for example, because the search target over proportionally matched the VWM item, or to refresh memory information for the later memory task—so that their attention was then biased towards the VWM matching items during search (Woodman & Luck, 2007). Another suggestion for explaining these contradictory findings is that transferring items into the passive state takes time, which might have been too short in some of the experiments that observed capture (Han & Kim, 2009). This is also in line with two recent studies arguing that template rejection might exist, but not as an early automatic attentional down-regulation process, but rather as a late voluntary compensative behavior after an initial attentional capture (Beck, Luck, & Hollingworth, 2018; Moher & Egeth, 2012).

The abovementioned studies about how VWM items might attract attention investigated whether any feature of a complete item stored in VWM can be ignored or used to reject distractors. This situation is different from the situation described at the beginning, in which a specific feature of a VWM template is defining the search target (cup’s shape), while another feature of the same cue object is search irrelevant (cup’s color) or might even define a search distractor. If VWM maintains objects in the form of bound features (Luck & Vogel, 1997; Marshall & Bays, 2013; O’Craven, Downing, & Kanwisher, 1999; Shen, Tang, Wu, Shui, & Gao, 2013), then all features of a currently important VWM template should be retained in an active format.

We recently showed within a highly controlled laboratory setting (reflecting the everyday example of the cup search described at the beginning of the paper) that it seems impossible to ignore the search-irrelevant feature of the VWM template and to selectively bias attention only towards objects matching the search-defining feature (Foerster & Schneider, 2018, 2019). Our experimental paradigm consisted of a VWM-based search task with a search target changing for each trial. Participants had to saccade quickly and accurately to a target object that was accompanied by a distractor (Foerster & Schneider, 2018). The saccade was used as a proxy for overt attention given that a covert shift of attention obligatorily precedes each saccade (Deubel & Schneider, 1996; Schneider, 1995). The target was defined by its shape-defined object identity (e.g., a pot) which was indicated prior to each search display by a colored, yet search-irrelevant cue (e.g., a red pot). Critically, the irrelevant color reappeared on the distractor shape (e.g., a red glass) in half of the trials, and participants were informed about this misleading nature of the color (Experiment 3). Nevertheless, participants saccaded more often to a distractor that matched the cue’s irrelevant color than to a distractor of another color. This effect has already been replicated and extended to the situation where the color is only an attribute of the object stored in long-term memory (LTM) rather than presented during search (Kerzel & Andres, 2020). Moreover, and in line with our results, objects in a search display matching a completely task-irrelevant color belonging to an object contained in VWM can be saccaded to faster and more accurately for a later second task, even in the absence of a distractor (Hollingworth, Matsukura, & Luck, 2013), and can bias the landing position of saccades in the global effect paradigm (Herwig, Beisert, & Schneider, 2010; Hollingworth et al., 2013), and the accuracy and latency of corrective saccades (Hollingworth & Luck, 2009; Hollingworth & Matsukura, 2019). In two letter report experiments, we could show that a color-matching distractor captures not only the eyes but also covert attention (Foerster & Schneider, 2019). Thus, in all these studies, visual attention is involuntarily biased towards the object that matches the VWM content in its irrelevant color, indicating that top-down control operates in an object-based and involuntary manner (i.e., top-down controlled attention biases by irrelevant features of a VWM object).

However, it is known that color is exceptionally effective in attracting attention (Hollingworth & Beck, 2016; Rutishauser & Koch, 2007; Soto et al., 2005; Soto et al., 2006; Williams, 1966, 1967), so that effects adhering to color might be dimension specific and do not necessarily generalize to other feature dimensions. Thus, the question remains whether the involuntary top-down bias might be limited to capture by color. Moreover, it is an open question as to why different features of VWM objects seem to elicit capture effects with different capture strength. One idea formulated in the scope of the abovementioned dual-task paradigm (search within the retention interval of a VWM task) is that the discriminability of target and distractor in the potentially capturing feature is essential (Duncan & Humphreys, 1989; Soto et al., 2005; Soto et al., 2006). In addition, we reasoned that the target–distractor similarity in not only the capturing irrelevant feature but also in the target-defining feature might be crucial. These hypotheses are in line with results from singleton search tasks (Barras & Kerzel, 2017a, b; Theeuwes, 1992). When searching for a target singleton from a specific feature dimension, capture strength by a singleton from another feature dimension can be influenced by the similarity of singletons and distractors in both dimensions. However, when searching for a singleton, the search target is defined by the difference between the target and distractors. Thus, it is not surprising that target–distractor similarity also influences singleton capture, given the well-established target–distractor similarity theories of biased competition (Bundesen, 1990; Duncan & Humphreys, 1989; Schneider, Einhäuser, & Horstmann, 2013). Even more important, searching repeatedly for a specific singleton is a very special case of visual search. In real-world tasks, we usually have one target at a time in mind (VWM) that we need to search for in order to accomplish a specific subtask. Moreover, we do not search for the same target again after having successfully found it and acted on it, but search for the next task-relevant target. Here, we present a series of experiments addressing the question of whether and how, in ecologically more valid searches for trial-by-trial changing targets (VWM targets), target–distractor similarity in the relevant as well as in the irrelevant feature determines attentional capture.

The results of eight experiments, following the basic paradigm of VWM-based search (Foerster & Schneider, 2018), show that the similarity of the target and the distractor with regard to their search-relevant as well as to their search-irrelevant feature determines whether attentional capture is strong enough to show up in overt behavior—here, in eye movements. Moreover, we demonstrate that color is not better than shape in capturing attention. By adjusting target–distractor similarity accordingly, we could successfully introduce or reduce oculomotor capture by color as well as by shape, mirroring the mechanism found in singleton search paradigms (Barras & Kerzel, 2017b; Theeuwes, 1992). Specifically, the bias by an irrelevant VWM feature (e.g., red) is stronger when the target and distractor are more distinct in terms of the search-irrelevant feature (e.g., red and blue instead of red and pink), and the more similar the target and distractor are in terms of the search-relevant feature (e.g., circle and blob instead of star and blob). Our findings show that this relation is independent of whether color or shape is the relevant feature, arguing for a general mechanism of involuntary object-based, top-down control by VWM templates. Thus, search-irrelevant features can involuntarily capture attention, and the capture strength depends on the target–distractor similarity of the environmental objects in the relevant and irrelevant feature dimensions. We conclude that this capture is only measurable in overt eye-movement behavior (the proxy for covert visual attention) if the relative bias signal of the irrelevant feature is high enough, which depends on the target-distractor similarity in all features.

## Experiment 1

In Experiment 1, we investigated whether distractors matching a search cue in its search-irrelevant shape will capture attention. On the basis of a paradigm from our previous study (Foerster & Schneider, 2018), we used colored real-world object shapes for the search task. However, this time, color defined the target, and shape had to be ignored. Specifically, the color of a real-world object informed participants at the beginning of each trial about the trial’s target color. The color varied in a trial-by-trial manner. After a variable duration fixation interval, a search display appeared consisting of a target and a distractor positioned to the left and right of fixation. Participants were asked to saccade to the colored object that matched the search cue’s color. This target never matched the object shape of the search cue and thus, identity. Instead, known by the participants, the distractor matched the cue’s object identity in half of the trials.

### Methods

#### Participants

A sample size of eight was chosen based on the results of our previous study (Foerster & Schneider, 2018). With an expected Cohen’s *d*_*z*_ around 2, an alpha level of .05, and a power of .99, the needed sample size is six. Two male and six female students from Bielefeld University, Germany, with a mean age of 25 years, ranging from 22 to 31 years, participated in the experiment after having provided written informed consent. In all experiments, participants reported normal or corrected-to-normal visual acuity, were naïve with respect to the study’s purpose, and were paid 8€ per hour of participation. All studies were approved by the Bielefeld University’s Committee for Ethics at the Department of Psychology.

#### Apparatus and stimuli

**Fig. 1:**
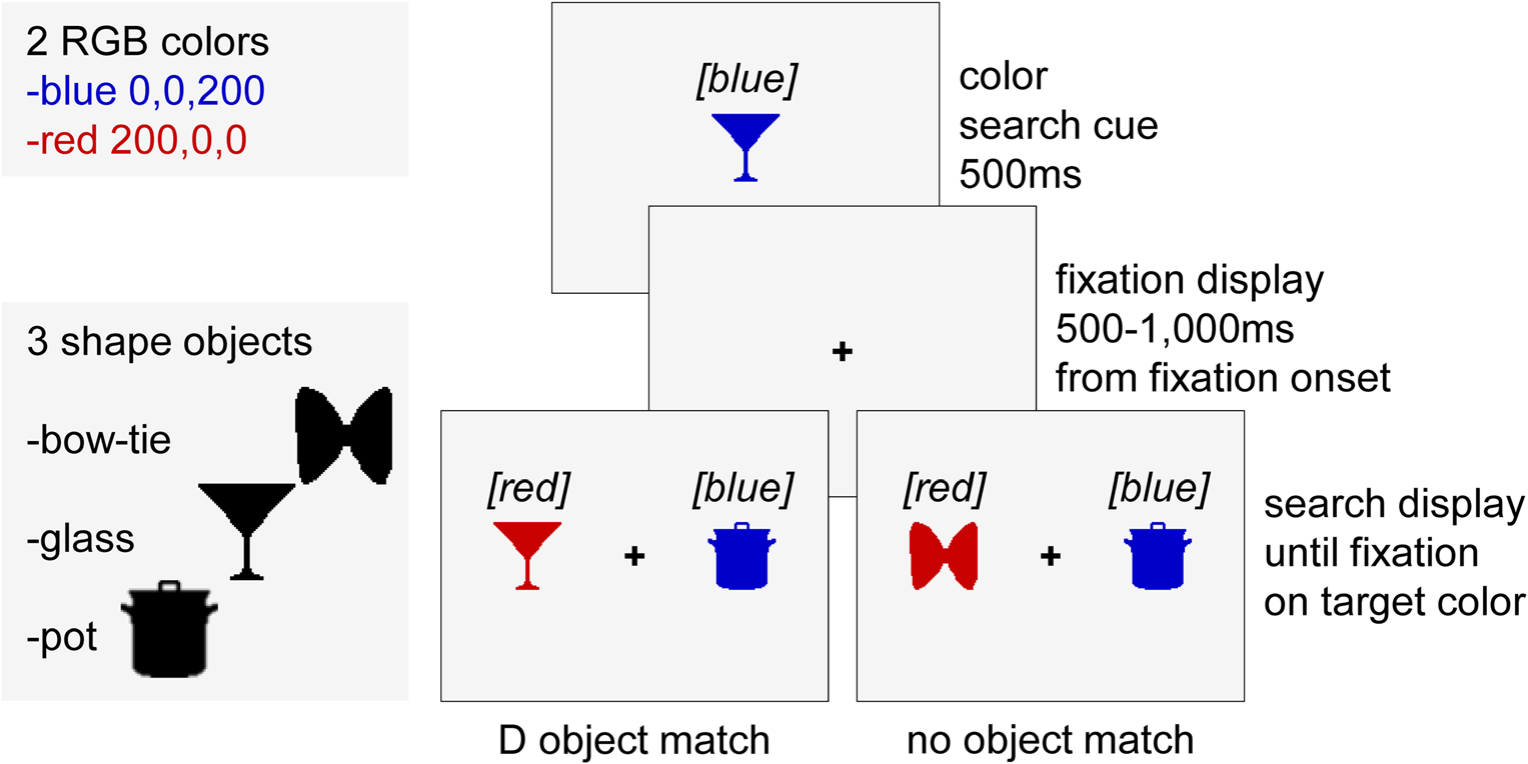
Material, procedure, and design of Experiment 1. Participants had to saccade to an object with a cued color. The target was always of another object identity than the search cue. The distractor was either of the same object identity as the cue (D object match) or had another object identity (no object match). The words in squared brackets are added for greyscale printing and were not present during the experiment. (Color figure online)

Stimuli were displayed on a 19-in. color monitor (View Sonic Graphics series G90fB, Brea, CA), 100 Hz, and a spatial resolution of 1024 × 768 pixels extending 36 × 27 cm. An EyeLink 1000 desktop eye tracker (SR Research, Canada) recorded participants’ right gaze position at 1000 Hz. A chin-and-forehead rest stabilized participants’ heads at a viewing distance of 71 cm. SR Research’s Experiment Builder software was used to program and run the experiment on a Dell Precision T3600 with an NVIDIA GeForce GTX 970 graphics card. Luminance and color of all presented stimuli were measured at screen center in CIE *L, x, y* coordinates with an X-Rite i1 Pro spectrophotometer. All stimuli were presented on a grey background (RGB: 245, 245, 245; *L* = 97 cd/m2, *x* = .3, *y* = .3). A black plus (+; RGB: 0, 0, 0; *L* = 0 cd/m^2^, *x* = .3, *y* = .3) with a size of 0.45 degrees of visual angle (°v.a.), was used as a central fixation marker. The shapes of a bow tie, a glass, and a pot could appear filled with either the color blue (RGB: 0, 0, 200; *L* = 9 cd/m^2^, *x* = .2, *y* = .1) or the color red (RGB: 200, 0, 0; *L* = 20 cd/m^2^, *x* = .6, *y* = .3) as search stimuli (see Fig. 1). The stimuli were modified objects obtained from http://cvcl.mit.edu/MM/objectCategories.html (Konkle, Brady, Alvarez, & Oliva, 2010). Stimuli were filled with one of the two (red or blue) colors and adjusted to a size of 49 × 49 pixels equaling 1.39° v.a. in foveal vision by using MATLAB R2013b. The search cue was always presented in the center of the screen. Search target and distractor were located 5.67° v.a. (200 pixels) to the left and right of the center.

#### Procedure

The experiment started with written instructions on the computer screen, followed by a nine-point eye-tracking calibration and validation procedure, and a practice trial that was not included in the analysis. The experiment consisted of 384 trials, separated in four blocks, and took about half an hour. After each block, a feedback display informed participants about the number of completed and total blocks. Participants could start each block by pressing the space bar. Each trial started with a central colored object presented for 500 ms, cueing the trial’s target color. Afterwards, participants had to keep fixation within an area of 2.5° v.a. around the central fixation (+) for a randomly chosen duration of between 500 ms and 1000 ms (uniform distribution). If the participant was unable to fixate for the randomly chosen duration (e.g., 500 ms) within a 5-s period, the trial was abandoned and repeated at a random position within the block. In addition, calibration was repeated in this case. Otherwise, the fixation period was followed by the search display, with one colored object appearing on the left side and another colored object appearing on the right side. The object presented in the same color as the search cue was the target, while the other colored object was the distractor. Participants were asked to make a fast and accurate saccade to the target-colored object. SR Research’s Experiment Builder software was used to detect saccade onsets online with a velocity threshold of 30°/s and an acceleration threshold of 8000°/s^2^. If the participants did not start a saccade within 400 ms from search stimuli onset, the trial was abandoned and repeated at a random position within the block. A fixation of at least 100 ms duration within an area of 2.8° v.a. around the target completed a trial. A high-pitched tone followed each trial in order to inform participants that they had completed the trial successfully and that the next trial was initiated.

#### Design

The experiment consisted of two match conditions (see Fig. 1). In the no-object-match condition (no object match), the cue, the target, and the distractor were of different object shapes and therefore identities. In the distractor object-match condition (D object match), the distractor and the search cue had the same object identity, while the target was of another object identity. All combinations of match conditions (2), locations (2), colors (2), and object combinations (6) were equally often completed per block in random order. The experiment consisted of four blocks of 96 trials each, adding up to 384 trials in total.

#### Analysis

Data were analyzed using R3.4.0 (R Development Core Team, 2016) and the packages plyr (Wickham, 2016) and BayesFactor (Morey, Rouder, Jamil, & Morey, 2015). Plotting routines of ggplot2 (Wickham, Chang,, & RStudio, 2016) and gridExtra (Auguie & Antonov, 2016) were used. The dependent variables were proportion and median latency of the first saccades landing at the target (tolerance diameter of 2.8° v.a. around the target). Paired *t* tests and Bayesian paired *t* tests were used to compare the dependent variables across the two conditions. In case of violation of a normal distribution according to the Kolmogorov-Smirnoff test, results were validated with Wilcoxon signed-rank tests. Only deviating nonparametric results are reported. A chance level of .05 was applied. A Bayes factor higher than 3 was interpreted as evidence for the alternative hypothesis that the conditions differ. A Bayes factor below 0.3 was interpreted as evidence for the null hypothesis that the conditions do not differ. All data are provided in the Supplementary Material. None of the studies reported here was preregistered.

### Results

**Fig. 2:**
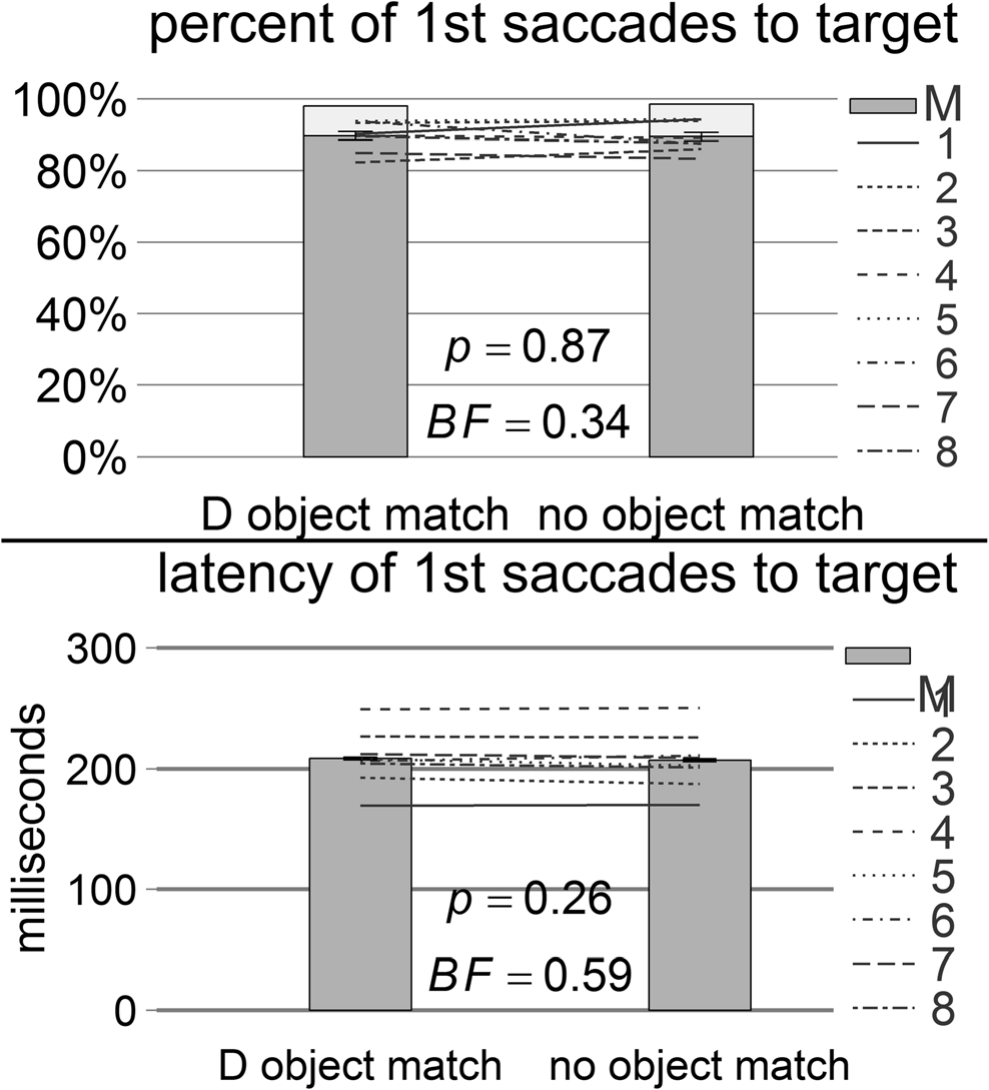
Results of Experiment 1. Percentage (top) and median latency (bottom) of all first saccades reaching the target area in case of the cue-distractor object match (D object match) and in case of different objects presented as search cue, target, and distractor (no object match). The lines represent individual subject data, and the dark-grey bars represent sample means of the individual data. The light-grey bars in the upper diagram represent the percentage of first saccades reaching the distractor area. Error bars correspond to standard errors of the mean of the paired differences across object-match conditions

On average, 1.8 trials per participant had to be repeated because central fixation was not kept for the specified duration. On average, 12.8 trials per participant had to be repeated because no saccade started within 400 ms from search stimuli onset. Two percent of all first saccades reached neither the target nor the distractor (tolerance diameter of 2.8° v.a.). This proportion was not significantly different across the object-match conditions, and there was no evidence for or against an effect in terms of the Bayes factor, *t*(7) = 1.28, *SE* = 0.46, *p* = .24, Cohen’s *d*_*z*_ = 0.45, *BF* = 0.63. About 90% of all participants’ first saccades landed directly on the target (see Fig. 2, top). This proportion was not significantly different across object-match conditions with an undecided Bayes factor, *t*(7) = 0.17, *SE* = 1.18, *p* = .87, Cohen’s *d*_*z*_ = 0.06, *BF* = 0.34. Finally, the median latency of these target saccades was on average 298 ms and not significantly different across object-match conditions, without evidence in any direction by the Bayes factor, *t*(7) = 1.22, *SE* = 1.23, *p* = .26, Cohen’s *d*_*z*_ = 0.43, *BF* = 0.59.

### Discussion

In contrast to our previous study (Foerster & Schneider, 2018), and in line with our target–distractor similarity considerations, we did not find oculomotor capture by a memory-matching distractor. The crucial difference to previous experiments is that, here, object shape/identity instead of color was the search-irrelevant and potentially capturing feature. However, as assumed in the literature, the discriminability of target and distractor in terms of the potentially capturing feature dimension might be essential (Soto et al., 2005; Soto et al., 2006). The used real-world objects have complex shapes that do not differ significantly when viewed in the periphery. Thus, their attentional bias might be so subtle that it does not show up in overt shifts of attention. The search-relevant colors, in contrast, appear highly dissimilar and will thus have high attentional bias signals, so that the bias signal of the relevant color should strongly overweigh a potential bias signal by the memory-matching complex shape. In Experiment 2, we investigated whether memory-matching search-irrelevant shapes will capture the eyes if the target and distractor shapes used appear highly dissimilar, while it is more difficult to discriminate target and distractor on the basis of the relevant color. In Experiment 3, we manipulated only the similarity in the relevant feature, and in Experiment 4, we manipulated only the similarity in the irrelevant feature, in order to see whether both modulate capture strength.

## Experiments 2–4

**Fig. 3:**
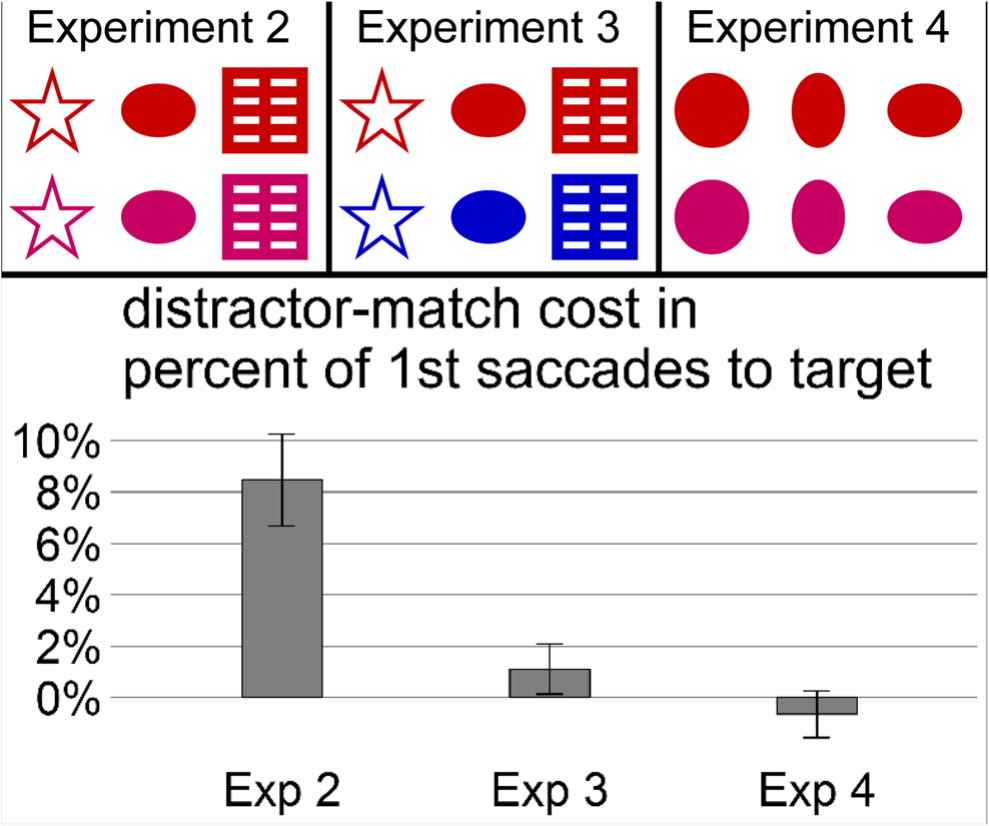
Upper part: Stimulus material of Experiments 2, 3, and 4. A star, a horizontal blob, and a square were used as irrelevant shapes in Experiments 2 and 3, while a circle, a vertical blob, and a horizontal blob were used as irrelevant shapes in Experiment 4. Stimuli appeared in red or pink relevant colors in Experiments 2 and 4, but in red or blue relevant colors in Experiment 3. The color feature (variation in rows in the upper graphs) was relevant for the search in all three experiments, while the shape feature (variation in columns in the upper graphs) was irrelevant in all three experiments. Lower part: Capture costs of Experiments 2, 3, and 4, operationalized as difference in percentage of first target saccades between the no shape match and the distractor shape match. Error bars correspond to standard errors of the means

In Experiments 2–4, participants had to saccade to a shape with a target color previewed by a search cue of another shape, which the distractor could inherit in half of the trials, as was known by the participant. However, in contrast to Experiment 1, abstract shapes were used instead of the real-world objects, in line with traditional stimuli classes used in visual-search studies (e.g., Wolfe, 1994). In addition, the following three experiments varied in how similar the used objects were in the relevant color and in the irrelevant shape, operationalized by using different sets of colors and shapes (see the upper part of Fig. 3). We hypothesized that target-distractor similarity in the relevant as well as in the irrelevant feature influences capture strength similarly, as demonstrated in the findings of the singleton capture literature (cf. Barras & Kerzel, 2017b; Theeuwes, 1992). Specifically, we hypothesized that high similarity in the relevant colors together with low similarity in the irrelevant shapes should elicit a shape-capture effect (Experiment 2). Moreover, enhancing the discriminability in the relevant colors (Experiment 3) as well as enhancing the similarity in the irrelevant shapes (Experiment 4) should reduce the capture effect significantly.

### Method

Three samples of eight students from Bielefeld University, Germany, with the same prerequisites as in the previous experiment, participated in the three experiments (Experiment 2: two males, six females; mean age of 24 years ranging from 19 to 28 years; Experiment 3: two males, six females; mean age of 25 years ranging from 21 to 28 years; Experiment 4: two males, six females; mean age of 24 years ranging from 20 to 29 years). In Experiment 2, one additional participant had been replaced because of chance-level performance even in the no-match baseline (50.5%).

Apparatus and stimuli were the same as in Experiment 1, except for two differences. This time, an EyeLink 1000 tower eye tracker (SR Research) recorded participants’ right eye movements while stabilizing participants’ head with the system’s inherent forehead and chin rest, again, at a viewing distance of 71 cm. More importantly, other stimulus sets were used. In Experiments 2 and 3, a star, a blob, and a square were used, which were assumed to be of low similarity and thus high discriminability due to the differences in roundness, edges, and horizontal and vertical lines that should elicit quite different neural processing (Habak, Wilkinson, Zakher, & Wilson, 2004; Held & Shattuck, 1971; Hubel & Wiesel, 1962; Poirier & Wilson, 2006). In Experiment 4, a circle, a vertical blob, and a horizontal blob were used, which were assumed to be highly similar—not only subjectively but also because they differ only in terms of curvature. In Experiments 2 and 4, stimuli were colored either red (RGB: 200, 0, 0; *L* = 20 cd/m^2^, *x* = .6, *y* = .3) or pink (RGB: 200, 0, 100; *L* = 11 cd/m^2^, *x* = .4, *y* = .2)—colors that are subjectively highly similar and also in proximal distance on any proposed color perception scheme (for a recent review of color perception, see Witzel & Gegenfurtner, 2018). In Experiment 3, stimuli were colored either red (RGB: 200, 0, 0; *L* = 20 cd/m^2^, *x* = .6, *y* = .3) or blue (RGB: 0, 0, 200; *L* = 9 cd/m^2^, *x* = .2, *y* = .1), so they are subjectively highly dissimilar and also far apart in distance on any proposed color perception scheme. Objective measures of our subjective similarity measures are delivered in our experiments by saccade-target discrimination performance (i.e., by the percentage of first saccades that went towards the target rather than towards the distractor) in the no-match baselines. All stimuli are displayed in Fig. 3 (upper part). The exact design of the three experiments can be seen in the Supplementary Material (Figs. S1, S3, and S5 in the individual effects file).

The procedure of each experiment was the same as in Experiment 1, except that the limit for a saccadic reaction was increased to 1500 ms from search onset, so that accuracy was further prioritized over saccade latency. In addition, a potential capturing effect by the search-irrelevant shape can, in this way, not be attributed to speed pressure or insufficient time for recoding shape as a template for rejection (Han & Kim, 2009). Each experiment consisted of two conditions (cf. Fig. 1). Either the search cue’s shape did not appear in the search display (no shape match), or the distractor matched the search cue in the irrelevant shape feature (D shape match).

A between-design analysis of variance (ANOVA) on the potential capture cost in terms of percentage of first target saccades (D match condition − no match condition) was conducted, with experiment as the between-subjects factor. Independent-samples *t* tests and Bayesian *t* tests compared the capture strength (D match condition − no match condition) of Experiment 2 to Experiment 3 as well as to Experiment 4. Corrected degrees of freedom were reported in case of Welch correction for the independent-samples *t* tests. Finally, paired *t* tests and Bayesian paired *t* tests per experiment compared the percentage of first target saccades and their median latencies across the two conditions, exactly as in Experiment 1, to reveal in which experiments significant capture could be found.

### Results

On average 2.8 trials in Experiment 2, 1.2 trials in Experiment 3, and 4.4 trials in Experiment 4 had to be repeated per participant because central fixation was not kept for the specified duration. In Experiments 2 and 3, one participant did not perform a saccade within the reaction time interval of 1500 ms in one of the 384 trials. In Experiment 4, 1.1 trials per participant had to be repeated because no saccade was executed within the reaction time interval. All experiments’ mean percentages of direct target saccades and their latencies in both conditions as well as their statistical comparison are listed in Table 1. Table 1 shows that participants were worse at discriminating red and pink objects than the same objects in red and blue (Experiment 2 vs. 3). This is in line with our judgement of higher similarity of the former.

**Table 1:** All experiments’ simple effects of the match manipulation

DV	Exp	*M*(D)	*M*(no)	*t*	*SE*	*p*	Cohen’s *d*_*z*_	*BF*
Percentage of direct target saccades	1	89.68	89.47	0.17	1.18	.87	0.06	0.34
	2	64.06	75.53	4.74	1.79	<.01	1.67	22.45
	3	90.43	91.54	1.13	0.98	.29	0.40	0.55
	4	80.40	79.75	0.73	0.89	.49	0.26	0.42
	5	69.08	80.66	4.06	2.85	<.01	1.44	11.68
	6	85.22	88.09	2.40	1.19	<.05	0.85	1.97
	7	80.27	87.24	4.72	1.48	<.01	1.67	22.11
	8	72.59	78.06	2.37	2.31	<.05	0.84	1.90
Latency [ms] of direct target saccades	1	208.61	207.12	1.22	1.23	.26	0.43	0.59
	2	252.22	240.81	2.23	5.12	.06	0.79	1.63
	3	207.69	209.41	1.04	1.65	.33	0.37	0.52
	4	228.54	227.01	0.92	1.65	.39	0.33	0.47
	5	260.33	249.85	2.43	4.31	<.05	0.86	2.04
	6	222.62	221.30	0.61	2.15	.56	0.22	0.39
	7	224.52	221.76	1.25	2.20	.25	0.44	0.61
	8	277.94	264.09	3.34	4.14	<.05	1.18	5.54

*Note.* DV = dependent variable; Exp = Experiment; *M*(D) = mean value of the distractor-match condition; *M*(no) = mean value of the no-match baseline; *t* = *t* value of the paired *t* test; *SE* = standard error of the mean paired differences; *p* = significance level of the paired *t* test; *BF* = Bayes factor of the Bayesian paired *t* test

The ANOVA on capture cost in terms of percentage of first target saccades with experiment as between-subject factor was significant, *F*(2, 21) = 14.20, *p* < .001, η^2^ = .57. This indicates that target-distractor similarity modulated capture strength (see Fig. 3). The capture cost (D match − no match) in Experiment 2 was significantly larger than in Experiment 3, *t*(10.84) = 3.61, *p <* .01, Cohen’s *d* = 1.81, *BF* = 13.16, and significantly larger than in Experiment 4, *t*(10.27) = 4.56, *p* < .01, Cohen’s *d* = 2.28, *BF* = 56.15, while capture cost of Experiments 3 and 4 did not differ significantly, *t*(13.88) = 1.33, *p* = .20, Cohen’s *d* = 0.67, *BF* = 0.76. Capture costs are presented in Fig. 3 (lower part). Paired *t* tests and Bayesian paired *t* tests per experiment comparing the two conditions (see Table 1) revealed that the shape-match effect was actually only significant in Experiment 2 (see also individual effect figures in the Supplementary Material, Figs. S2, S4, and S6).

### Discussion

In contrast to Experiment 1, a cue-shaped distractor decreased the proportion of first saccades to the color-defined target compared with a differently shaped distractor in Experiment 2. This is a proof that oculomotor capture by a search-irrelevant shape of a search cue is possible. Moreover, whether attentional capture by a search-irrelevant shape shows up in overt shifts of attention depends on target-distractor similarity. Oculomotor capture was observed when target and distractor are highly discriminable in the potentially capturing search-irrelevant feature dimension and highly similar in the search-relevant feature dimension (Experiment 2). Capture was impeded when stimuli were more discriminable in their search-relevant color (Experiment 3) as well as when stimuli were more similar in their search-irrelevant shape (Experiment 4). We think that oculomotor capture results from a stimulus set in which the bias signal by the search-irrelevant feature is strong enough to overrule the bias signal by the search-relevant feature. High target-distractor similarity in a feature dimension reduces the difference signal elicited by this feature, while high target-distractor dissimilarity in a feature dimension enhances the difference signal elicited by this feature. If this is a general priority control mechanism (Schneider et al., 2013), then the same modulation should hold for color rather than shape being the irrelevant feature. The following set of four experiments (5–8) will investigate whether this assumption is valid.

## Experiments 5–8

Experiments 5–8 were conducted to demonstrate that the effects of target-distractor similarity in the relevant as well as irrelevant feature on oculomotor capture generalize to the case where shape defines the search target and color is irrelevant.

### Method

Four samples of eight students from Bielefeld University, Germany, with the same prerequisites as in the previous experiments participated in the experiment (Experiment 5: three males, five females; mean age of 24 years, ranging from 18 to 33 years; Experiment 6: two males, six females; mean age of 24 years, ranging from 22 to 27 years; Experiment 7: two males, six females; mean age of 25 years, ranging from 21 to 28 years; Experiment 8: one male, seven females; mean age of 23 years, ranging from 20 to 26 years).

Apparatus and procedure were the same as in the previous experiments, except that now the shape of the search cue defined the search target, while the other shape was the distractor, so that participants were asked to make a fast and accurate saccade to the target shape. Again, the experiments consisted of two conditions (see Figs. S7, S9, S11, S13 in the Supplementary Material). In the no color-match condition (no color match), the cue, the target, and the distractor appeared in different colors. In the distractor color-match condition (D color match), the distractor and the search cue had the same color, while the target had another color. All combinations of conditions (2), locations (2), shapes (2), and color combinations (6) were equally often completed per block, in random order.

**Fig. 4:**
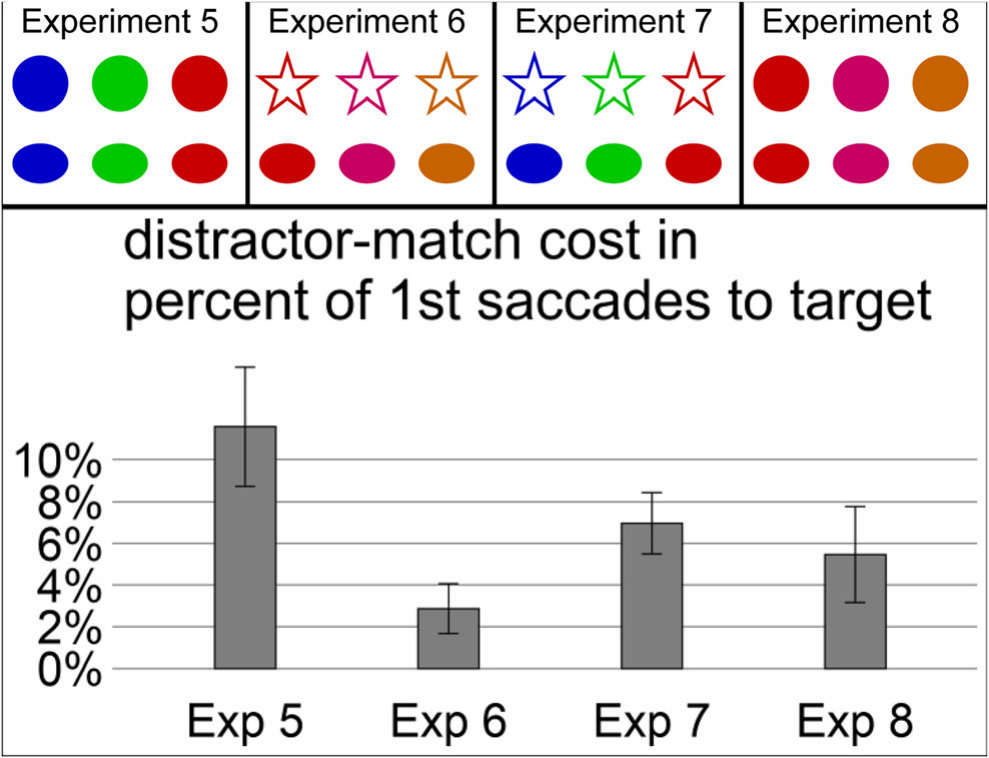
Upper part: Stimulus material of Experiments 5, 6, 7, and 8. A circle and a horizontal blob were used as relevant shapes in Experiments 5 and 8, while a star and a horizontal blob were used as relevant shapes in Experiments 6 and 7. Stimuli appeared in blue, green, or red irrelevant colors in Experiments 5 and 7, but in red, pink, or orange irrelevant colors in Experiments 6 and 8. The shape feature (variation in rows in the upper graphs) was relevant for the search in all three experiments, while the color feature (variation in columns in the upper graphs) was irrelevant in all three experiments. Lower part: Capture costs of Experiments 5, 6, 7, and 8, operationalized as difference in percentage of first target saccades between the no color match and the distractor color match. Error bars correspond to standard errors of the means

Different sets of stimuli were used. A circle or a horizontal blob could appear in Experiments 5 and 8, which were highly similar because they only vary in curvature. In Experiments 6 and 7, a star and a horizontal blob were used, which were more discriminable due to the difference in edges and horizontal and vertical lines (Habak et al., 2004; Held & Shattuck, 1971; Hubel & Wiesel, 1962; Poirier & Wilson, 2006). These shapes appeared in the highly distinct colors blue (RGB: 0, 0, 200; *L* = 9 cd/m^2^, *x* = .2, *y* = .1), green (RGB: 0, 200, 0; *L* = 58 cd/m^2^, *x* = .3, *y* = .6), or red (RGB: 200, 0, 0; *L* = 20 cd/m^2^, *x* = .6, *y* = .3) in Experiments 5 and 7, and in the highly similar colors red (RGB: 0, 0, 200; *L* = 9 cd/m^2^, *x* = .2, *y* = .1), pink (RGB 200, 0, 100; *L* = 11 cd/m^2^, *x* = .4, *y* = .2), or orange (RGB: 200, 100, 0; *L* = 41 cd/m^2^, *x* = .5, *y* = .4) in Experiments 6 and 8. All stimuli are shown in Fig. 4 (upper part). The exact design of the three experiments can be seen in the Supplementary Figs. S7, S9, S11, and S13. Data analysis was the same as in the previous set of experiments.

We predicted that oculomotor capture by a distractor matching the irrelevant color of the cue would be stronger in Experiment 5 than in the other three experiments due to its ideal combination of high similarity in the relevant shape and high dissimilarity in the irrelevant color. However, capture strength should be smallest in Experiment 6 with highly dissimilar relevant shapes and highly similar irrelevant colors.

### Results

On average 2.9 trials per participant in Experiment 5, 4.0 trials in Experiment 6, 0.5 trials in Experiment 7, and 6.2 trials in Experiment 8 had to be repeated because central fixation was not kept for the specified duration. In Experiment 5, one further trial of two participants had to be repeated because no saccade was executed within the reaction time interval of 1500 ms. On average, 0.8 trials per participants in Experiment 6 and 0.5 trials in Experiment 8 had to be repeated for this reason. In Experiment 7, one participant did not execute a saccade within the reaction time interval in only one trial. All experiments’ mean percentages of direct target saccades and their latencies in both conditions as well as their statistical comparison are listed in Table 1. Table 1 shows that participants were worse at discriminating a blob from a circle than from a star of the same color (Experiments 5 vs. 7 and 6 vs. 8). This is in line with our judgement of higher similarity between a blob and a circle compared with a blob and a star.

The ANOVA on capture cost in terms of percentage of first target saccades, with experiment as a between-subjects factor, was significant, *F*(3, 28) = 3.14, *p <* .05, η^2^ = .25. Thus, target-distractor similarity seems to modulate the strength of capture by the irrelevant color. Capture cost (D match − no match) in Experiment 5 was significantly larger than the cost in Experiment 6, *t*(9.38) = 2.82, *p* < .05, Cohen’s *d* = 1.41, *BF* = 4.20, when both features were modulated and numerically larger than the cost in Experiment 7, *t*(10.50) = 1.44, *p* = .17, Cohen’s *d* = 0.72, *BF* = 0.84, and Experiment 8, *t*(13.42) = 1.67, *p* = .12, Cohen’s *d* = 0.83, *BF* = 1.04. Capture cost of Experiment 6 was significantly smaller than capture cost of Experiment 7, *t*(13.41) = 2.16, *p* < .05, Cohen’s *d* = 1.08, *BF* = 1.79, but only numerically smaller than of Experiment 8, *t*(10.49) = 1.00, *p* = .34, Cohen’s *d* = 0.50, *BF* = 0.60. Capture cost of Experiments 7 and 8 were not significantly different from each other, *t*(11.90) = 0.55, *p* = .60, Cohen’s *d* = 0.27, *BF* = 0.47. Capture costs are presented in Fig. 4 (lower part). Interestingly, the color capture cost in Experiment 5 was not significantly different from the shape capture cost in Experiment 2, but with undecided Bayes factors, *t*(11.76) = 0.93, *p* = .37, Cohen’s *d* = 0.46, *BF* = 0.57. Effects of the two conditions on the percentage of first target saccades and their latencies in each experiment are displayed in Table 1 and in the Supplementary Material (Figs. S8, S10, S12, and S14).

### Discussion

In Experiment 5, we could successfully replicate the oculomotor capture by the search-irrelevant color of a VWM object that we have reported in a previous publication (Foerster & Schneider, 2018). Together with the results of Experiment 2, this shows that capture can be found irrespective of whether shape or color is used as the relevant or irrelevant feature. The strength of this oculomotor capture does not necessarily differ, arguing that color is not better in capturing attention than shape (cf. Theeuwes, 1992). Crucially, the result patterns of Experiments 5–8 demonstrate that the color-capture effect is independently modulated by target-distractor similarity in the relevant and irrelevant feature, just like the shape-capture effect. Specifically, oculomotor capture by a search-irrelevant color can successfully be reduced by making the search objects highly dissimilar in the search-relevant shapes, and making the search objects highly similar in the search-irrelevant colors (Experiment 6). Manipulating only one dimension decreases the capture cost at least numerically (Experiments 7 and 8). Thus, the same factors that were responsible for the reduced capture by shape in Experiments 1, 3, and 4 reduced the color capture in Experiments 6–8.

## General discussion

Searching for a trial-wise varying target in visual-search tasks requires that objects in the environment that match the search target (probably retained as a template in VWM) are preferentially processed and selected. This can be achieved by setting a task-driven top-down control signal that gives higher attentional weights to objects matching the VWM template (Bundesen, 1990; Conci et al., 2013; Desimone & Duncan, 1995; Folk et al., 1992; Hollingworth, 2012; Wolfe, 1994; Woodman & Chun, 2006) compared with objects that do not match the VWM content. In most experimentally studied visual-search scenarios and many real-life situations, one specific feature of the VWM template is target defining, while other features are uninformative or even shared with distractors. Conflicting results have been reported for different visual-search paradigms (Foerster & Schneider, 2018; Gao et al., 2016; Olivers et al., 2006; Sala & Courtney, 2009; Soto & Humphreys, 2009).

Here, we tried to reconcile these opposing findings on whether it is possible to ignore search-irrelevant features of a VWM template object or whether such features will automatically capture attention. On the basis of the idea that target-distractor discriminability should play a key role in VWM-based capture (Barras & Kerzel, 2017b; Soto et al., 2005; Soto et al., 2006; Theeuwes, 1992), we investigated oculomotor capture by a search-irrelevant VWM feature in eight experiments with varying target-distractor similarity.

Taken all the experiments together, we can safely conclude that an irrelevant color as well as an irrelevant shape of a varying search target can capture the eyes. Crucially, the data of our eight experiments support our key hypothesis that target-distractor similarity is the decisive variable to predict the strength of the oculomotor capture by a VWM matching distractor. High similarity of target and distractor in the search-irrelevant feature reduces oculomotor capture (Experiments 1, 4, 6, and numerically in Experiment 8). However, high dissimilarity of target and distractor in the search-relevant feature reduces oculomotor capture by the irrelevant feature (Experiments 1, 3, 5, and numerically in Experiment 7). These two rules of thumb are valid, irrespective of whether color or shape is the relevant or irrelevant feature, indicating that capture by color does not adhere to a different mechanism than capture by shape. Instead, the typically used colors seem to be much more discriminable (red, blue, green, yellow) for the visual system than the typically used shapes (square, triangle, octagon, circle) are, and therefore set a stronger attentional bias signal. Specifically, if the attentional bias set to the irrelevant feature is stronger than the attentional bias set to the relevant feature, then behavioral effects of attentional capture will be strong (e.g., in Experiments 2 and 5).

We argue that relevant as well as irrelevant features of a VWM-template object involuntarily bias attention in an object-based, top-down controlled fashion. Whether this attentional bias can be measured as a capture effect (e.g., in terms of prolonged search times and/or increased fixations on the distractor) depends on the difference in the strength of the bias by the irrelevant feature compared with the relevant feature. The bias strength of a feature becomes higher with higher dissimilarity of target and distractors in this feature. Thus, observable capture by the irrelevant feature is fostered by high target-distractor dissimilarity in the irrelevant feature and high target-distractor similarity in the relevant feature. Future studies using EEG and relying on attentional components such as N2pc might be able to clarify whether a bias towards matching distractors can be tracked in situations in which no significant behavioral effect is observed. By centralizing either the target or the distractor, while keeping the other lateralized, it could be investigated whether the distractor-match versus no-match conditions differ in the N2Pc signal to the lateralized item (Barras & Kerzel, 2017a; Eimer & Kiss, 2010; Hickey, McDonald, & Theeuwes, 2006; Kumar, Soto, & Humphreys, 2009), even in situations without a behavioral difference.

How do our results relate to the singleton-capture literature? Our results are in line with results obtained with the additional singleton search paradigm (Barras & Kerzel, 2017a, b; Theeuwes, 1991; van Zoest, Donk, & Theeuwes, 2004). In this paradigm, participants search for a target singleton of a specific feature (e.g., color) in a search display that contains a target which they have to report (e.g., a bar with a certain orientation to report). The search display contains an additional singleton of another feature (e.g., shape) in some trials. Importantly, capture by the additional singleton is influenced by target-distractor similarity in the target-defining (task-relevant) feature as well as in the potentially capturing (task-irrelevant) feature. In Theeuwes (1992), for instance, finding a green circle singleton among green squares was slowed by a red square distractor singleton, while no capture by the color singleton was observed when both colors were yellowish, which produces high similarity in the irrelevant potentially capturing feature. However, finding a yellowish green circle singleton among yellowish red circles was slowed by a yellowish-red square, while no distraction by the shape singleton was observed with red and green items (i.e., high dissimilarity in the relevant feature). Thus, color capture was modulated by target-distractor similarity in the irrelevant feature (color), while shape capture was modulated by target-distractor similarity in the relevant feature (color). Shape similarity was not manipulated in this study. In Barras and Kerzel (2017a, b), participants reported the orientation of a line in a shape singleton, a red square, that appeared either among red circles or red diamonds. A green color singleton captured attention more strongly in the condition with low compared to high target-distractor similarity in the task-relevant shape feature. Van Zoest et al. (2004) showed that oculomotor capture by a tilted bar among vertical bars is more frequent the more tilted it is (i.e., the more it differs from the other items).

When searching for a singleton, the search condition is per definition based on a difference between target and distractors in one or several feature dimensions. Thus, it is not surprising that target-distractor similarity influences singleton capture in some way because it modulates the salience of the singletons (target or distractor). Although the target feature was blocked in the reported studies, so that feature search was possible, it is not clear whether participants actually used a feature or a singleton search mode (Bacon & Egeth, 1994). Moreover, searching for a singleton is a very special case of visual search that is seldom required in real-world tasks. Usually, we do not search for an object that is different from all other objects in the environment in a single feature. Instead, we have a specific target object in mind that we search for because it is relevant for an ongoing task. In addition, we do not continue to search for the same object once we have found it (LTM-based search; Kang & Woodman, 2014). Instead, we usually perform multistep tasks. Different objects are relevant at different time steps so that we have to search for them sequentially—a typical feature of many everyday tasks (e.g., when making a cup of tea; Land & Tatler, 2009). Thus, it is highly important to know how target-distractor similarity influences attentional guidance in this ecologically valid VWM-based visual-search scenario investigated here. Fortunately, the result pattern is in line with most singleton-search studies. Attention towards objects with task-irrelevant features (that are part of a VWM template) is stronger the less similar the objects are in this feature dimension, and the more similar the objects are in the target-defining dimension. Thus, relevant as well as irrelevant features of the VWM-template object seem to set a bias signal.

Note that in our studies, both the relevant and irrelevant features belong to a single object in VWM. When the relevant and irrelevant features belong to different objects, only objects in a search display matching the irrelevant feature/object in VWM capture attention if the search target is constant over trials, so that this relevant feature can be recoded into a resource-free long-term memory template and thereby making the irrelevant feature/object the only item in VWM (Kerzel & Witzel, 2019).

By which mechanism might relevant and irrelevant features of the VWM template object bias attention? The results could be explained within the biased competition framework of the theory of visual attention (TVA; Bundesen, 1990; Bundesen & Habekost, 2008). According to the TVA, attentional weights are calculated for each environmental object based on their feature salience and feature relevance. Specifically, objects that contain task-relevant features will be weighted higher. In addition, features that have high sensory evidence (e.g., a stronger contrast to the background) will have higher weights. Moreover, by multiplication of the sensory evidence that an object belongs to a category and the relevance (pertinence) value of this category, the attentional weight will be higher the stronger the evidence is that the object has a task-relevant feature. Hence, a blue object has a lower weight than a pink object, when red is task relevant. That all objects within an eye fixation compete for attentional selection is implemented in TVA in the fact that all relative attentional weights sum up to one. In classical TVA, this assumption holds for all objects within a single fixation (Bundesen, 1990), while an extension across fixations has been proposed (Poth & Schneider, 2018; Schneider, 2013). This competitive processing assumption of TVA and its extensions predicts that when red is task-relevant, a red object will get lower attentional weights when it is presented along a pink object than along a blue object, as the blue object produces less sensory evidence to be red than the pink object, and will thus be a weaker competitor. Extending classical TVA (Bundesen, 1990), we assume here that the highest weighted object should become the target for an eye movement (Carbone & Schneider, 2010; Schneider, 2013; Wischnewski, Belardinelli, Schneider, & Steil, 2010), thereby producing the oculomotor capture observed here. However, not only TVA and its extensions can explain the result pattern. In general, the data are perfectly in line with all other theoretical approaches that assume competition for a limited capacity of attentional allocation based on a mixture of bottom-up and top-down feature weighting of all environmental objects, such as the guided search theory (Wolfe, 2007) or other priority map models (Fecteau & Munoz, 2006; Itti & Koch, 2001; Schneider et al., 2013). Although all these theories would assume that capture strength is modulated by target-distractor similarity, this aspect was consistently neglected in behavioral experiments on attentional capture (for an exception, see Gaspelin, Ruthruff, & Lien, 2016).

Our assumption on the decisive role of the target-distractor similarity in the relevant and the irrelevant feature dimension and relative strength of feature bias signals should, for instance, also be informative when considering opposing results of the VWM literature on whether currently irrelevant VWM content biases attention during interim search tasks (Olivers et al., 2011, for a review). In these dual-task studies, VWM objects relevant for a later memory recognition task did sometimes slow search performance when appearing as distractors in an interim search task (Downing, 2000; Soto et al., 2005; Soto et al., 2008; Soto et al., 2006) and could sometimes effectively be ignored (Downing & Dodds, 2004; Woodman & Luck, 2007). It was assumed that whether capture is found or not depends on whether the VWM content is kept in an active or passive format (Olivers et al., 2006). As the VWM content in these dual-task studies does not define the search target, it can, in principle, be shifted into a passive format and reactivated only later for the memory retrieval part. Whether the VWM content is still active or already passive during the search is thought to depend on factors such as the time span between memory encoding and search (Han & Kim, 2009), or whether participants try to find relations between the two tasks (Woodman & Luck, 2007). The passive versus active distinction does not make predictions about the effects of similarity. However, it would predict that in our single-task design, in which the visual cue object has to be kept in an active format in VWM for the only ongoing search task, VWM-matching content should be in an active format and thus capture attention. Nevertheless, even in our paradigm, distractors matching the search-irrelevant feature of the search cue did not always attract the eyes more strongly than neutral distractors. The strength of the oculomotor capture was successfully predicted by the target-distractor similarity in the relevant as well as in the irrelevant feature. Thus, target-distractor similarity is an additional and maybe the crucial source that predicts whether and how strongly VWM-matching features will capture attention. The characteristics of the stimuli in the dual-task studies also differed, so the varying target-distractor similarity might have played an additional causal role for the conflicting results there. Indeed, studies that reported color capture often used highly distinctive colors, such as red, green, blue, and yellow (Soto et al., 2005; Soto et al., 2008; Soto & Humphreys, 2009; Soto et al., 2006). However, in one study, no color capture was found with those colors (Woodman & Luck, 2007). With highly complex and not very distinct shapes, no shape capture was observed (Downing & Dodds, 2004). Target-distractor similarity might not be the sole reason for the opposing results in the dual-task line of research of VWM-based capture. However, as target-distractor similarity is very likely an additional and crucial influencing factor in these dual-task paradigms, it should be considered in future attempts.

Taking target-distractor similarity in relevant and irrelevant features into account is also important when trying to compare the potential for attentional capture and guidance across feature dimensions. Investigations often found beneficial guidance by color compared with size, orientation, or shape (Rutishauser & Koch, 2007; Williams, 1967). However, these studies did not use varying exemplars of each dimension, and did not match the baseline discriminability of all features. We suggest that it is not the feature dimension, but the discriminability based on the exact features used, that determines the strength of voluntary and involuntary attentional guidance.

In conclusion, when searching for a trial-by-trial varying object that should be kept as a VWM template, attention is biased in a top-down fashion not only towards objects matching the search-defining feature of the template but also towards objects matching its search-irrelevant features. The strength of this involuntary top-down bias and whether it causes observable capture behavior depends on how similar the target is to surrounding objects in terms of search-defining as well as search-irrelevant features. This suggestion is in line with singleton-capture results, arguing for a general principle of attentional guidance.

## Electronic supplementary material

ESM 1 (ZIP 5436 kb)
