# Supplementary material for: Oculomotor capture by search-irrelevant features in visual working memory: on the crucial role of target–distractor similarity
Source: Atten Percept Psychophys. 2020 Mar 12;82(5):2379–92. doi: 10.3758/s13414-020-02007-0 (PMC7343749; doi:10.3758/s13414-020-02007-0)
Supplement: Supplementary file 1 — (ZIP 5436 kb) [file 13414_2020_2007_MOESM1_ESM.zip › SupplementaryMaterial/IndividualEffects.pdf]

Supplementary Material

The role of target-distractor similarity for oculomotor capture by search-irrelevant features in visual working memory

Rebecca M. Foerster & Werner X. Schneider

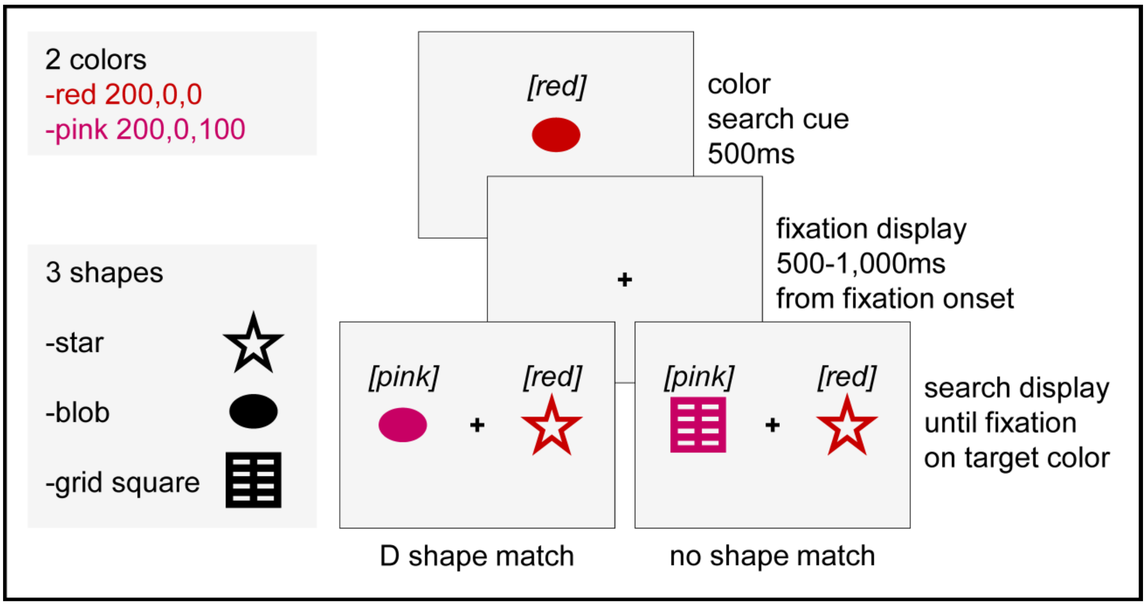

Figure S1. Material, procedure, and design of Experiment 2. Participants had to saccade to a shape with a cued color. The shape of the target was always different from the shape of the search cue. The shape of the distractor either matched the shape of the search cue (D shape match) or not (no shape match). The color words in squared brackets are added for greyscale printing and were not present during the experiment.

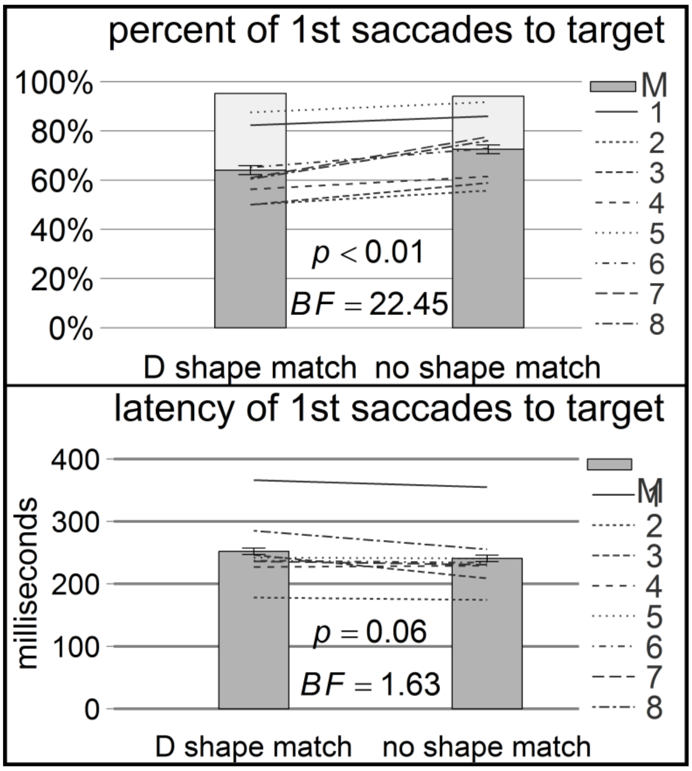

Figure S2. Results of Experiment 2. Percent (top) and median latency (bottom) of all first saccades reaching the target area in case of the cue-distractor shape match (D shape match) and in case of distinct shapes of cue, target, and distractor (no shape match). The lines represent individual subject data, and the dark-grey bars represent sample means of the individual data. The light-grey bars in the upper diagram represent the percentage of first saccades reaching the distractor area. Error bars correspond to standard errors of the mean of the paired differences across shape-match conditions

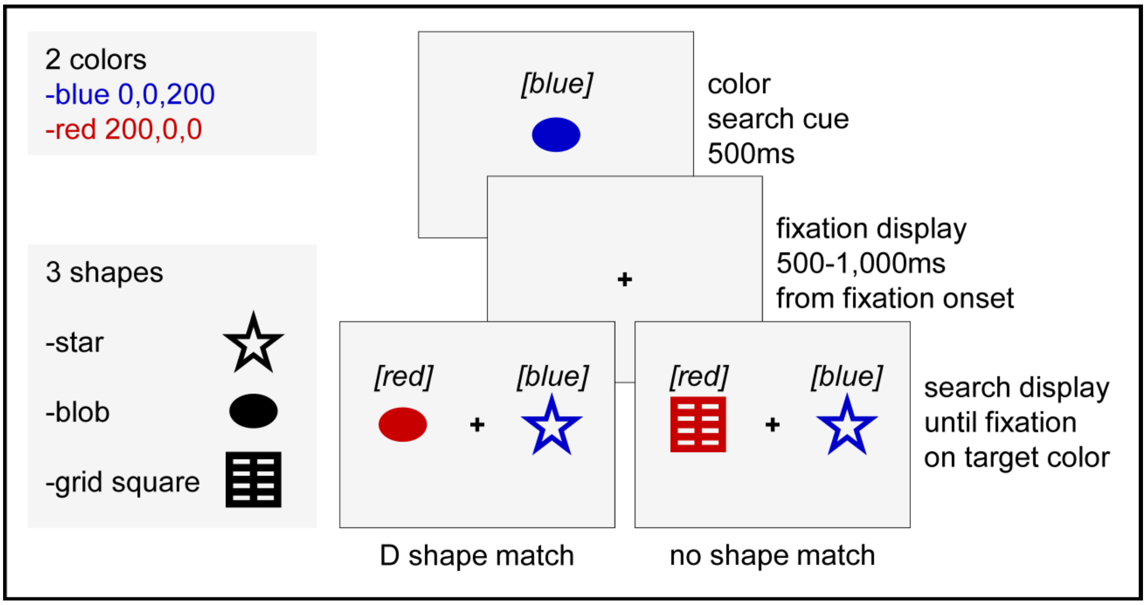

Figure S3. Material, procedure, and design of Experiment 3. Participants had to saccade to a shape with a cued color. The shape of the target was always different from the shape of the search cue. The shape of the distractor either matched the shape of the search cue (D shape match) or not (no shape match). The color words in squared brackets are added for greyscale printing and were not present during the experiment.

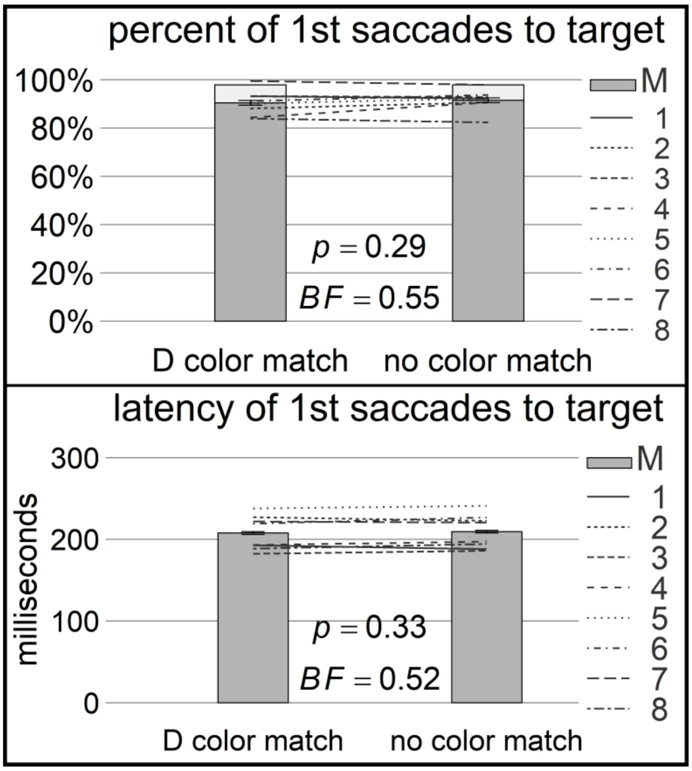

Figure S4. Results of Experiment 3. Percent (top) and median latency (bottom) of all first saccades reaching the target area in case of the cue-distractor shape match (D shape match) and in case of distinct shapes of cue, target, and distractor (no shape match). The lines represent individual subject data and the dark-grey bars represent sample means of the individual data. The light-grey bars in the upper diagram represent the percentage of first saccades reaching the distractor area. Error bars correspond to standard errors of the mean of the paired differences across shape-match conditions.

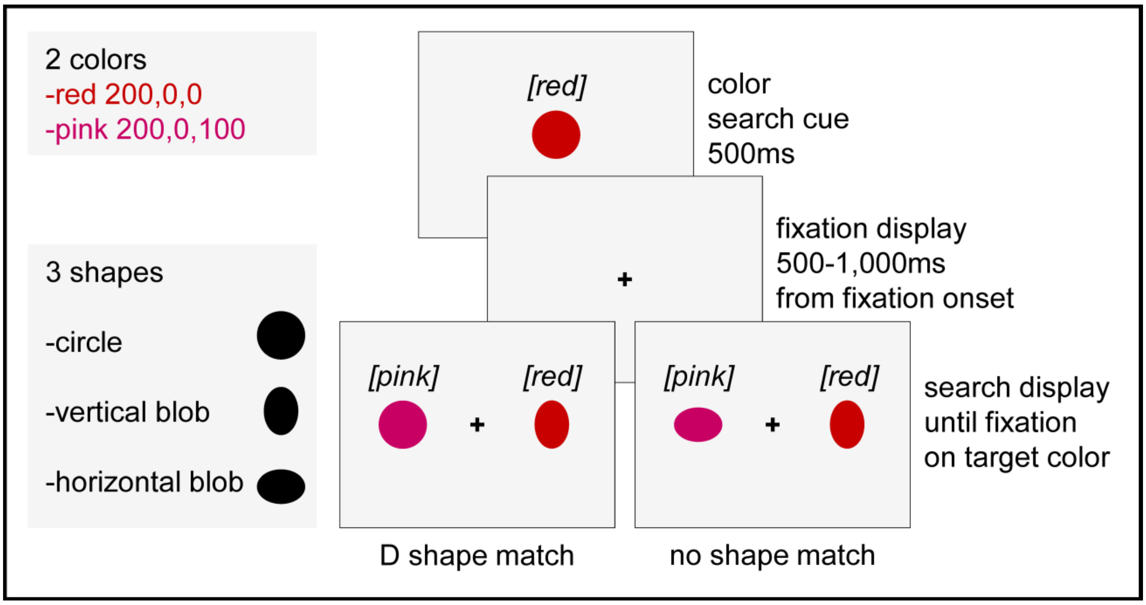

Figure S5. Material, procedure, and design of Experiment 4. Participants had to saccade to a shape with a cued color. The shape of the target was always different from the shape of the search cue. The shape of the distractor either matched the shape of the search cue (D shape match) or not (no shape match). The color words in squared brackets are added for greyscale printing and were not present during the experiment.

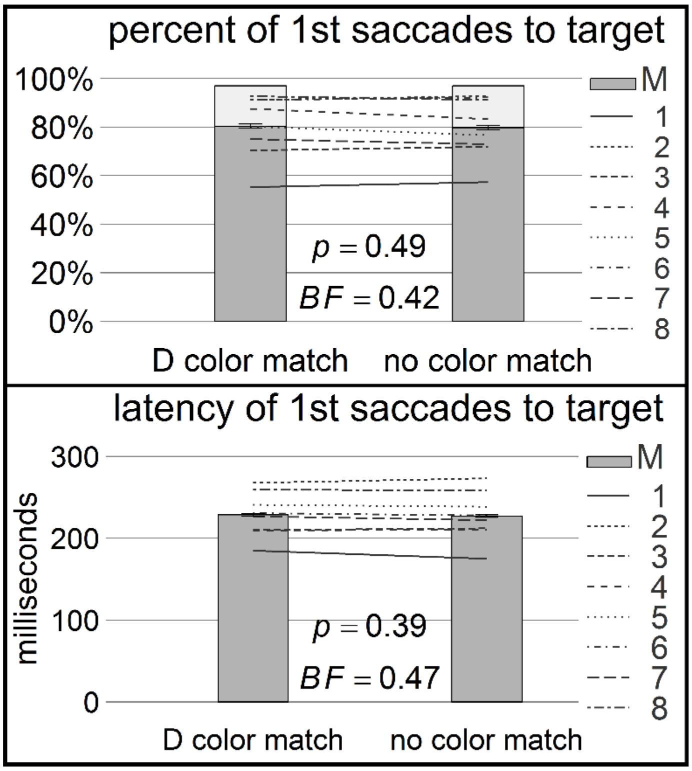

Figure S6. Results of Experiment 4. Percent (top) and median latency (bottom) of all first saccades reaching the target area in case of the cue-distractor shape match (D shape match) and in case of distinct shapes of cue, target, and distractor (no shape match). The lines represent individual subject data and the dark-grey bars represent sample means of the individual data. The light-grey bars in the upper diagram represent the percentage of first saccades reaching the distractor area. Error bars correspond to standard errors of the mean of the paired differences across shape-match conditions.

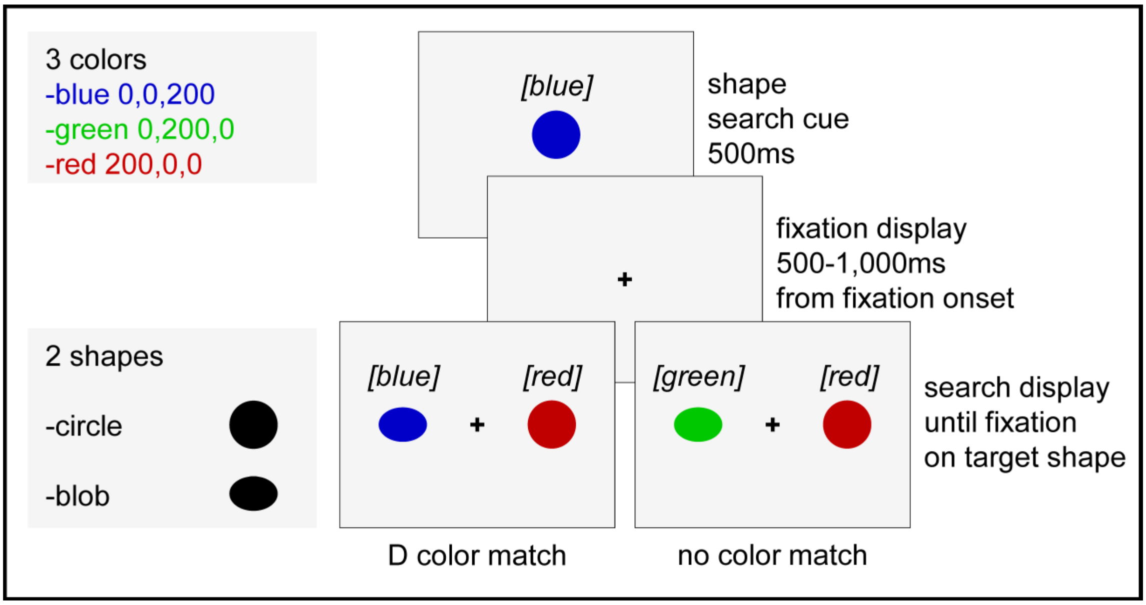

Figure S7. Material, procedure, and design of Experiment 5. Participants had to saccade to a cued target shape. The color of the target was always different from the color of the search cue. The color of the distractor either matched the color of the search cue (D color match) or not (no color match). The color words in squared brackets are added for greyscale printing and were not present during the experiment.

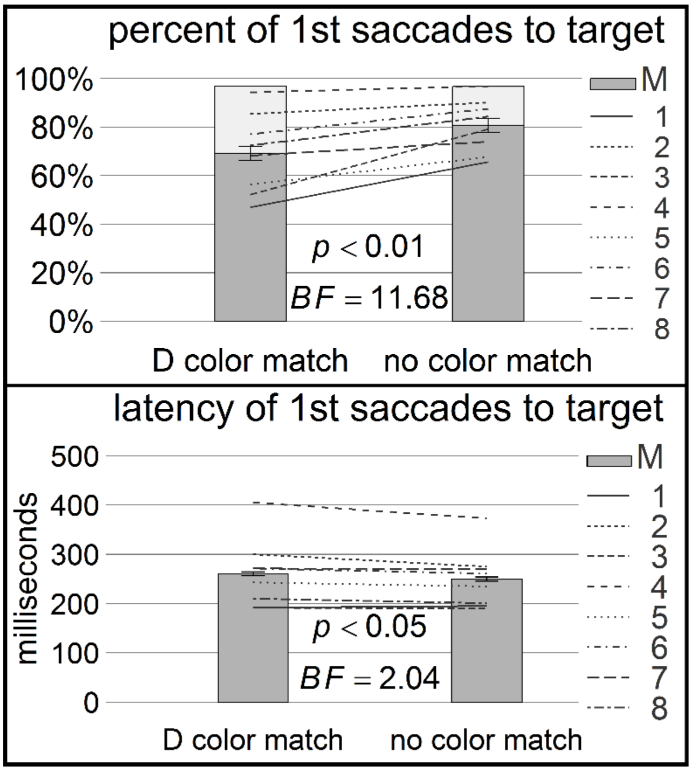

Figure S8. Results of Experiment 5. Percent (top) and median latency (bottom) of all first saccades reaching the target area in case of the cue-distractor color match (D color match) and in case of distinct colors of cue, target, and distractor (no color match). The lines represent individual subject data, and the dark-grey bars represent sample means of the individual data. The light-grey bars in the upper diagram represent the percentage of first saccades reaching the distractor area. Error bars correspond to standard errors of the mean of the paired differences across color-match conditions.

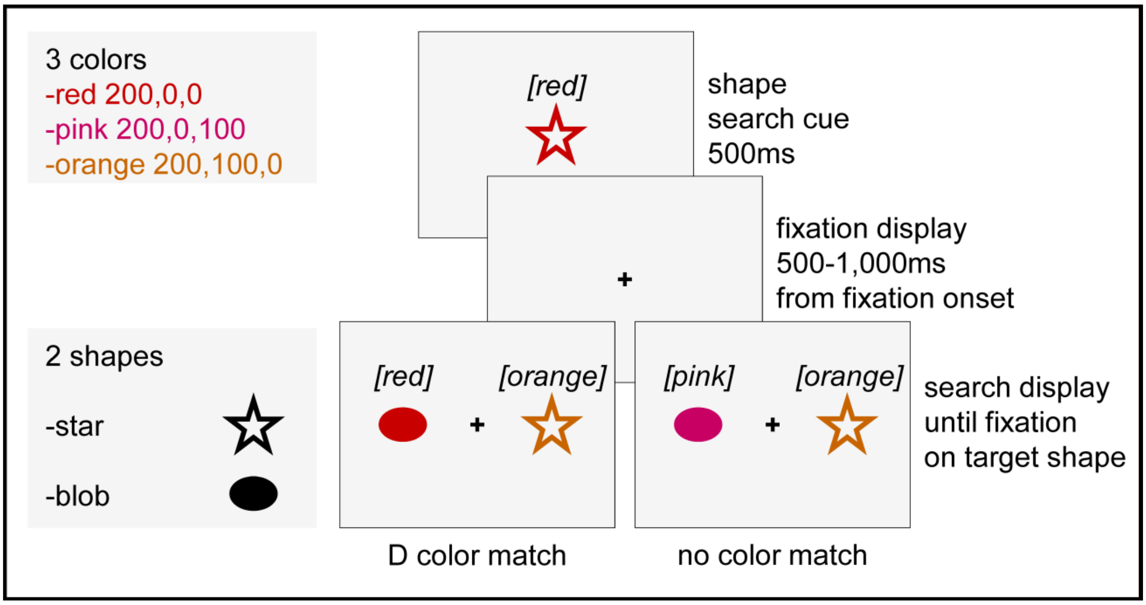

Figure S9. Material, procedure, and design of Experiment 6. Participants had to saccade to a cued target shape. The color of the target was always different from the color of the search cue. The color of the distractor either matched the color of the search cue (D color match) or not (no color match). The color words in squared brackets are added for greyscale printing and were not present during the experiment.

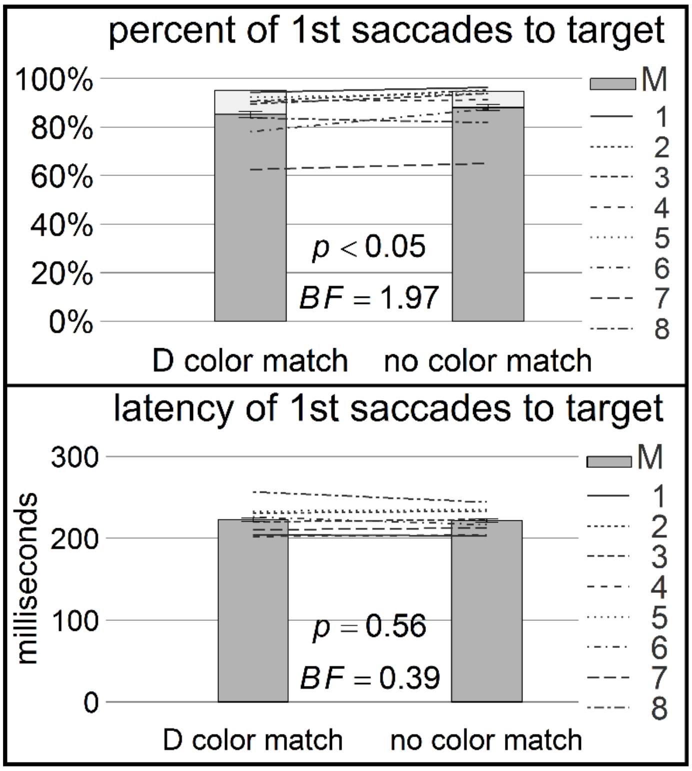

Figure S10. Results of Experiment 6. Percent (top) and median latency (bottom) of all first saccades reaching the target area in case of the cue-distractor color match (D color match) and in case of distinct colors of cue, target, and distractor (no color match). The lines represent individual subject data and the dark-grey bars represent sample means of the individual data. The light-grey bars in the upper diagram represent the percentage of first saccades reaching the distractor area. Error bars correspond to standard errors of the mean of the paired differences across color-match conditions.

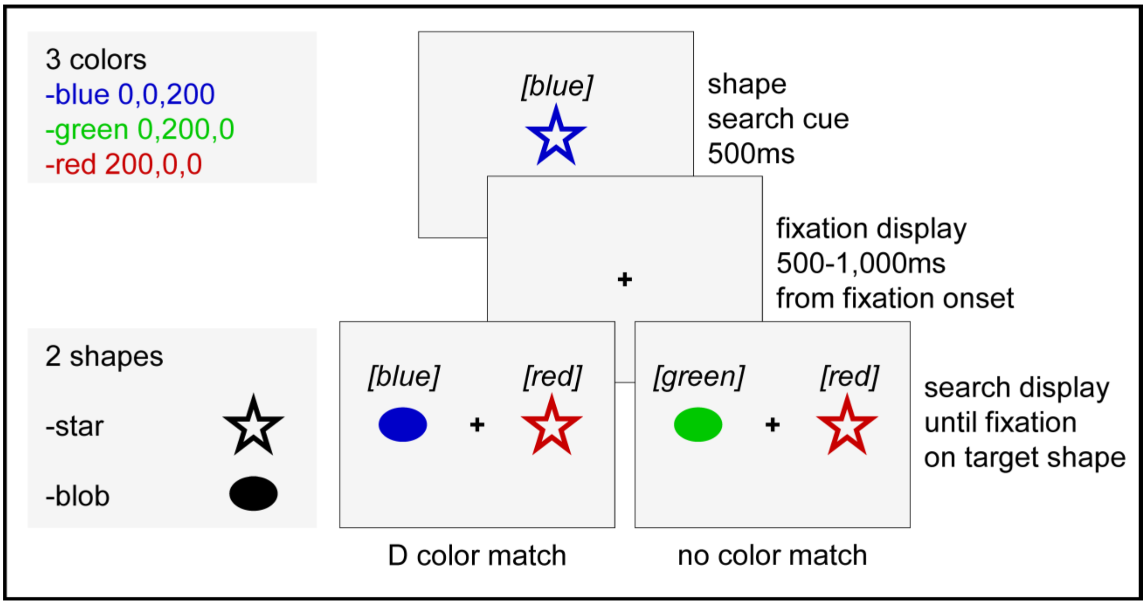

Figure S11. Material, procedure, and design of Experiment 7. Participants had to saccade to a cued target shape. The color of the target was always different from the color of the search cue. The color of the distractor either matched the color of the search cue (D color match) or not (no color match). The color words in squared brackets are added for greyscale printing and were not present during the experiment.

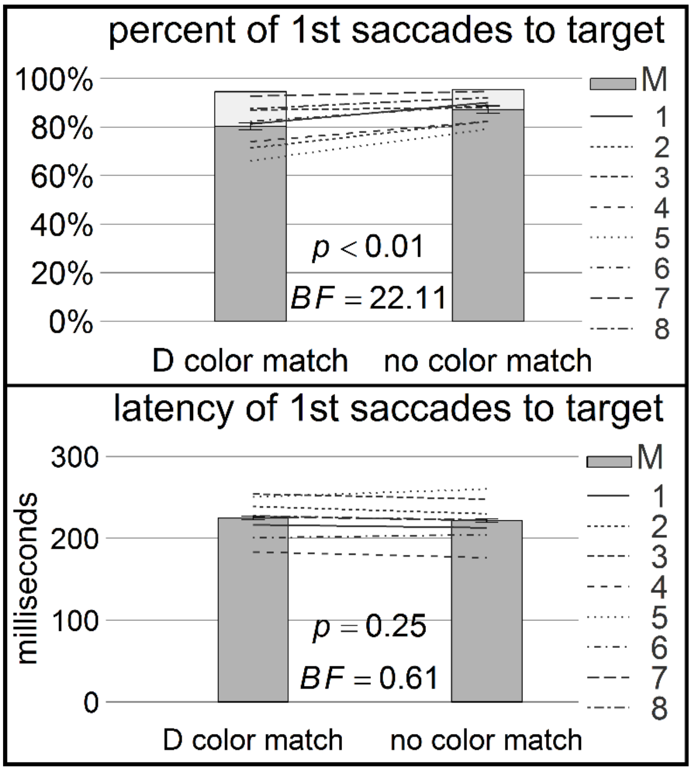

Figure S12. Results of Experiment 7. Percent (top) and median latency (bottom) of all first saccades reaching the target area in case of the cue-distractor color match (D color match) and in case of distinct colors of cue, target, and distractor (no color match). The lines represent individual subject data and the dark-grey bars represent sample means of the individual data. The light-grey bars in the upper diagram represent the percentage of first saccades reaching the distractor area. Error bars correspond to standard errors of the mean of the paired differences across color-match conditions.

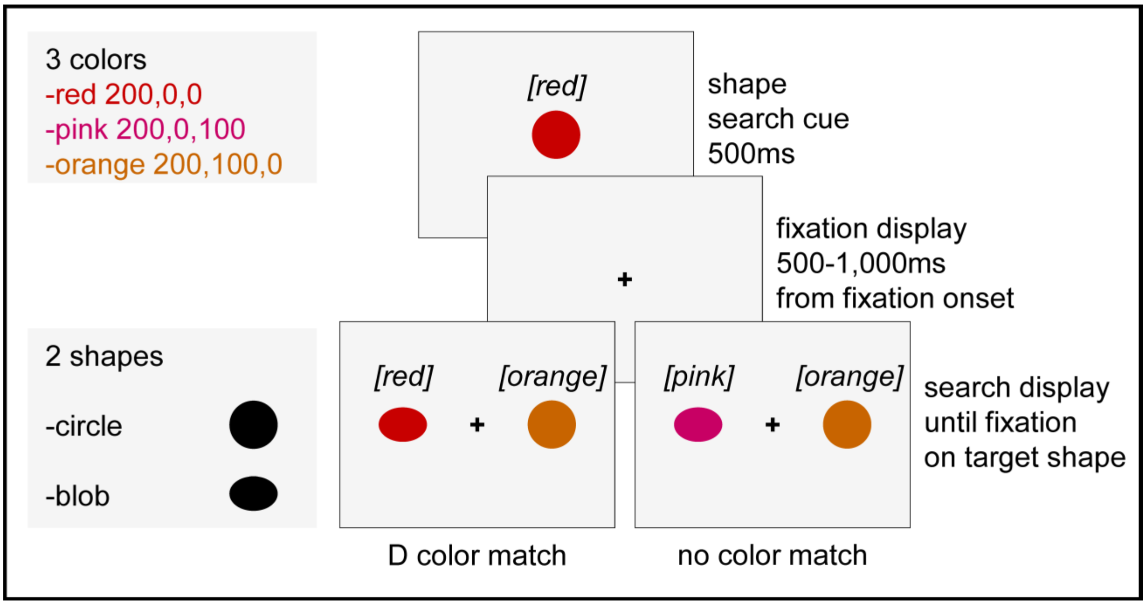

Figure S13. Material, procedure, and design of Experiment 8. Participants had to saccade to a cued target shape. The color of the target was always different from the color of the search cue. The color of the distractor either matched the color of the search cue (D color match) or not (no color match). The color words in squared brackets are added for greyscale printing and were not present during the experiment.

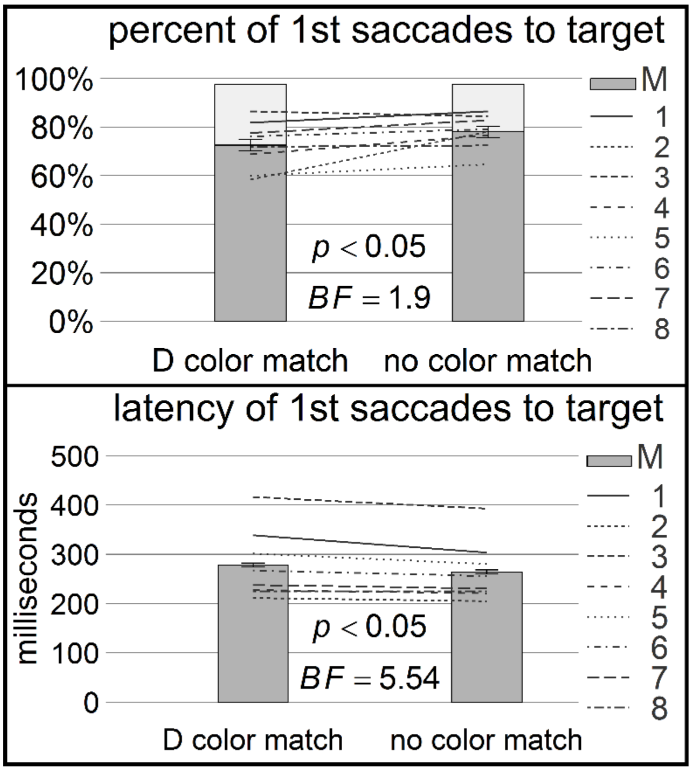

Figure S14. Results of Experiment 8. Percent (top) and median latency (bottom) of all first saccades reaching the target area in case of the cue-distractor color match (D color match) and in case of distinct colors of cue, target, and distractor (no color match). The lines represent individual subject data, and the dark-grey bars represent sample means of the individual data. The light-grey bars in the upper diagram represent the percentage of first saccades reaching the distractor area. Error bars correspond to standard errors of the mean of the paired differences across color-match conditions.
